# A rare case of congenital plexiform fibrohistiocytic tumor of the foot in a 4-year-old boy: case report and literature review 

**DOI:** 10.1080/23320885.2021.1986049

**Published:** 2021-10-01

**Authors:** Efterpi Demiri, Eleni Georgiadou, Olga-Christina Goula, Sofia-Eleni Tzorakoeleftheraki, Eleni Karagergou, Prodromos Hytiroglou

**Affiliations:** a Department of Plastic Surgery, School of Medicine, Faculty of Health Sciences, Aristotle University of Thessaloniki, Papageorgiou Hospital, Periferiaki Odos Neas Efkarpias, Thessaloniki, Greece; b Department of Pathology, School of Medicine, Faculty of Health Sciences, Aristotle University of Thessaloniki, Thessaloniki, Greece

**Keywords:** Plexiform fibrohistiocytic tumor, congenital soft-tissue tumors

## Abstract

The plexiform fibrohistiocytic tumor (PFHT) is an infrequent soft-tissue neoplasm with uncertain biological behavior. We report a rare congenital PFHT case in a 4-year-old boy, treated with wide excision and skin grafting. After a 52-month follow-up, no recurrence, regional or distant metastases were documented. A literature review on the management of PFHTs is reported.

## Introduction

Fibrohistiocytic tumors of the skin comprise a heterogeneous group of dermal and subcutaneous tumors of mesenchymal origin which show fibroblastic and myofibroblastic differentiation [1]. Plexiform fibrohistiocytic tumor (PFHT) or plexiform fibrous histiocytoma is considered a rare tumor of this group, characterized by borderline biologic behavior [2]. PFHT was first reported as a distinctive entity in 1988 by Enzinger and Zhang, who described a series of 65 cases in children and young adults, with a predilection for females [3]. This tumor most commonly presents as a slowly growing, asymptomatic nodule or, less frequently, as an indurated flat plaque, mainly located on the upper extremities (40–50%), followed by the lower extremities (9–20%), the trunk (8–15%) and the head and neck region (7–10%) [3–5]. PFHT is usually an acquired tumor, being more common in children and young adults under 20 years old; however, the tumor has also been described in infants and children less than 12 months of age [6].

In this article, we describe a very rare case of a congenital PFHT in a 4-year-old child, which is one of the very few cases of congenital PFHTs that have been reported in the literature so far [6–10]. Our aim is to describe the clinical and histological features of this tumor, present our treatment approach and results, and review the published literature on acquired and congenital PFHTs.

## Case presentation

**Figure 1.**
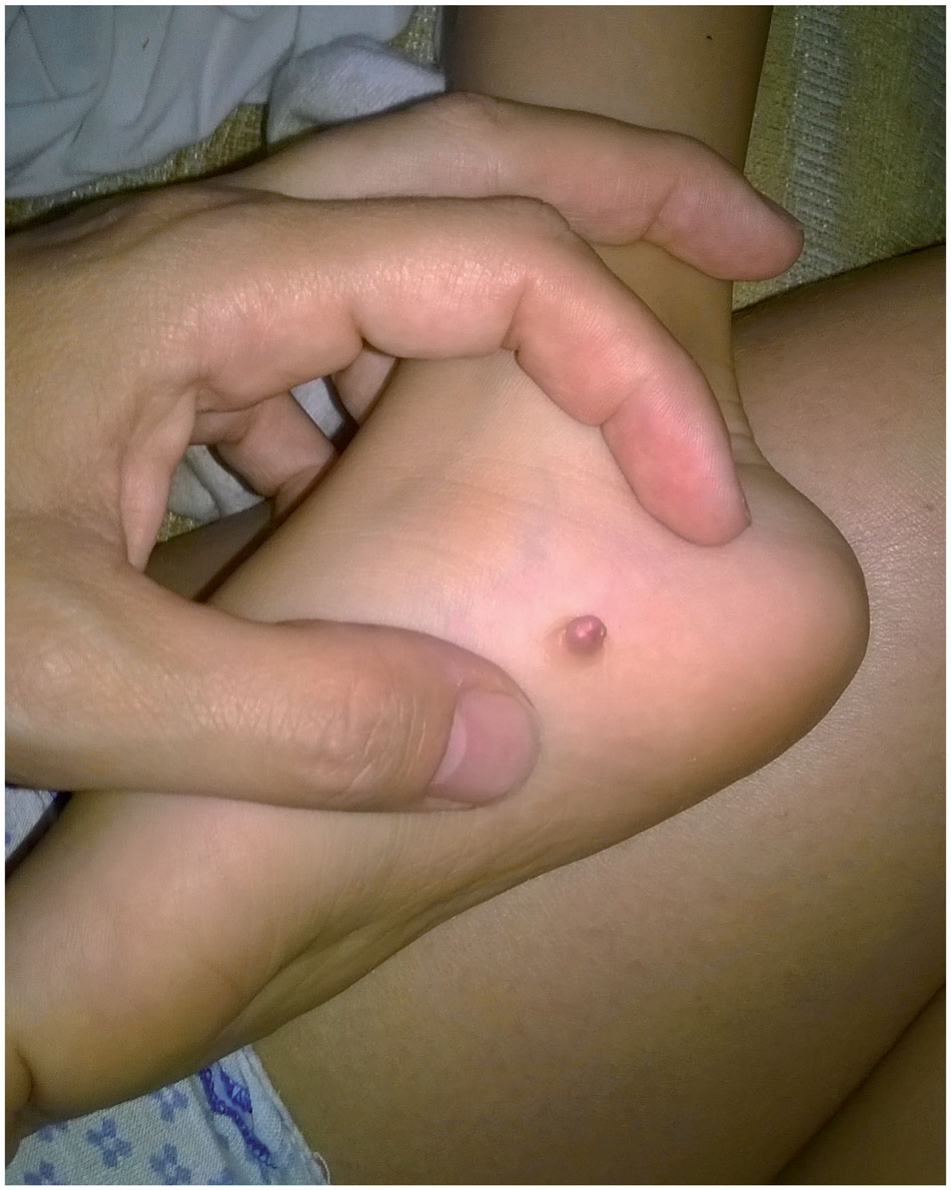
Clinical appearance of a solid rubbery nodular tumor on the right foot arch, before the initial surgical excision.

A 4-year-old boy presented with his parents at the Outpatient Clinics of our Department, following resection of a congenital nodule from the arch of his right foot two months earlier, elsewhere. According to the history, this skin lesion was present since birth as a macroscopically solid, asymptomatic and painless nodule that slowly increased in size, measuring –before excision- about 0.8 mm in diameter (Figure 1).

**Figure 2.**
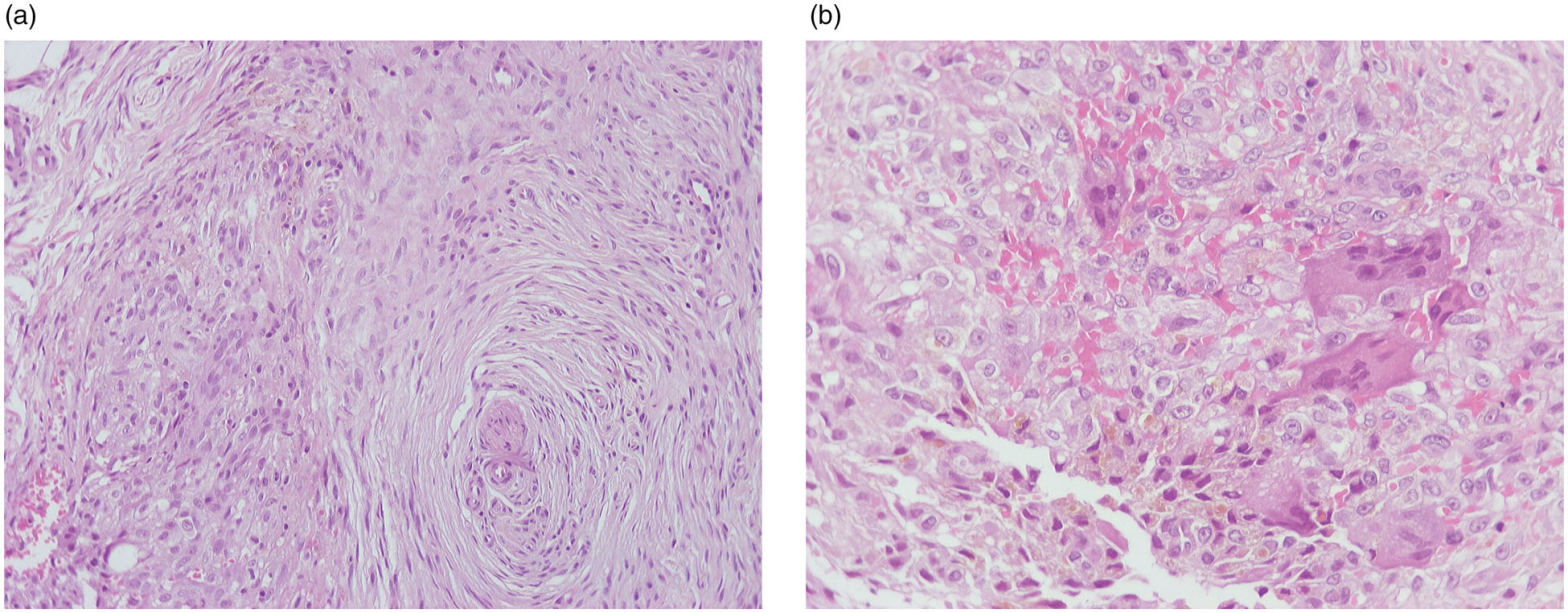
Histologic features: elongated neoplastic cells arranged in interconnected fascicles with a plexiform pattern (A). Ovoid and round neoplastic cells intermixed with osteoclast-like giant cells and hemosiderin deposition (B). (A: hematoxylin-eosin, ×100, B: hematoxylin-eosin, ×200)

The histopathological evaluation of the specimen revealed a dermal and subcutaneous spindle cell neoplasm with moderate cellularity. The majority of the cells were elongated, with eosinophilic cytoplasm and ovoid nuclei with minimal pleomorphism, arranged in interconnected fascicles with a plexiform pattern. Intermixed with these cells, there was a small subset of medium-sized, ovoid or round cells. Their cytoplasm was eosinophilic and the nuclei were ovoid, with mild pleomorphism and distinct nucleoli. These cells were arranged in clusters along with a few osteoclast-like giant cells and hemosiderin deposition (Figure 2). There was no mitotic activity. The lesion extended to the surgical margins. On immunohistochemical stains, the neoplastic cells were immunoreactive for vimentin, CD34 and CD10. The medium-sized, ovoid and round cells and the osteoclast-like giant cells were positive for CD68. A diagnosis of PFHT was made on the basis of the histologic features.

**Figure 3.**
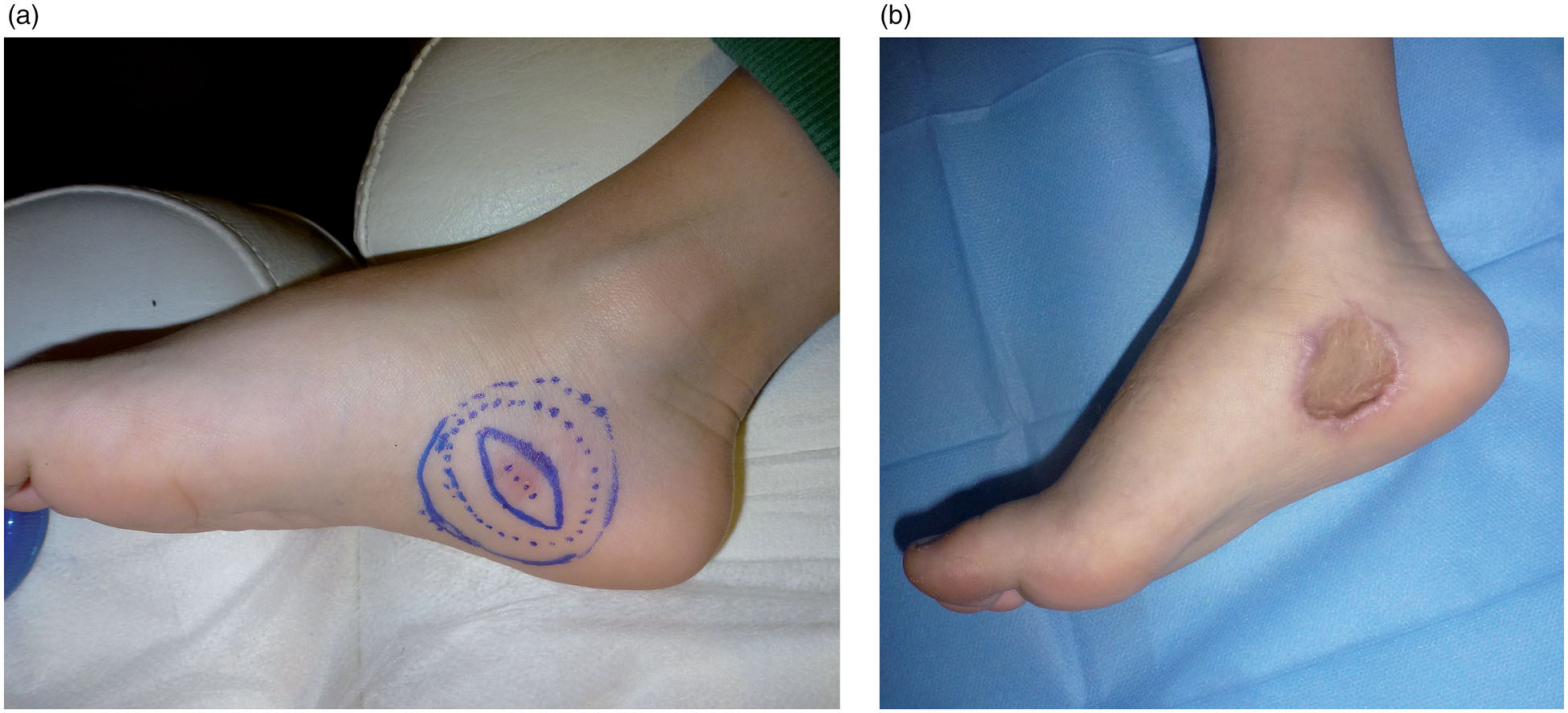
Preoperative view and design of the excision with 2 cm margins (A). Early postoperative result after skin graft coverage of the defect (B).

Due to positive margins, a wider excision was performed under general anesthesia, with 2 cm excisional margins around the existing scar and down to the underlying muscle fascia. The defect was covered with a full-thickness skin graft harvested from the left groin area. The immediate postoperative period and wound healing of both operated areas on the foot and the donor site was uneventful (Figure 3). The pathology report following re-excision was negative for residual tumor.

**Figure 4.**
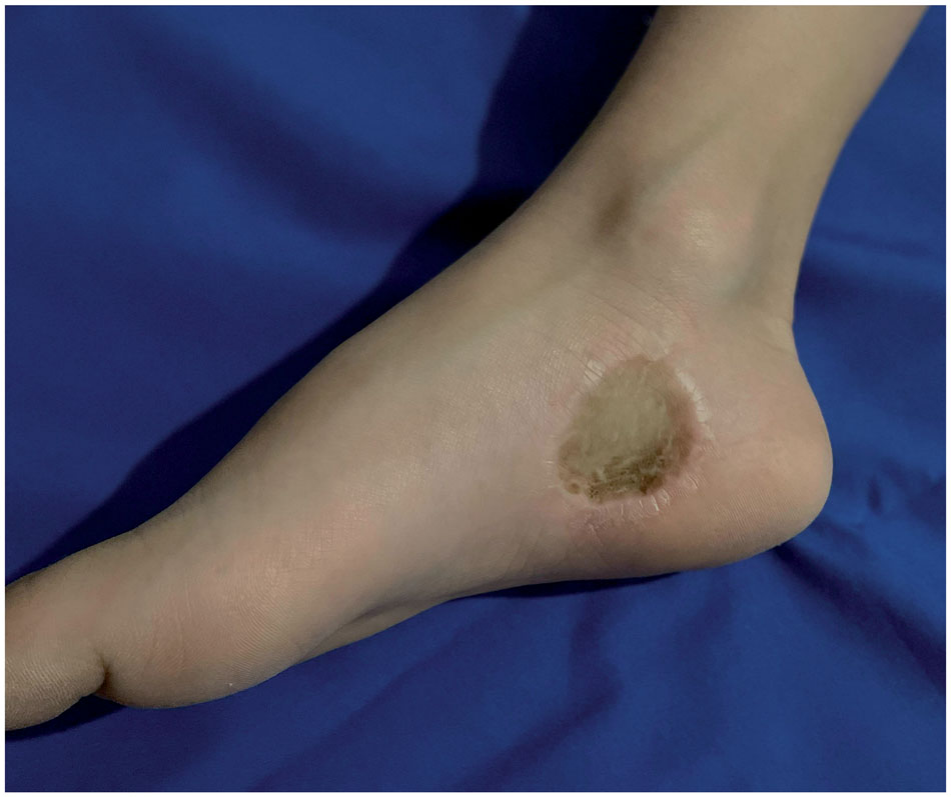
Long-term postoperative view of the operated area 52 months after the wide excision and skin grafting.

Approximately four months later, the patient presented with palpable lymph nodes in both groin areas. An ultrasound scan of both inguinal sites was performed reporting enlarged lymph nodes, which showed reactive features and were negative for tumor. On follow-up, seven months postoperatively, the clinically palpable groin lymph nodes subsided and a computed tomography scan of the thorax, abdomen, and pelvis showed no pathology. On 52 months’ follow-up, there was no evidence of local recurrence, regional lymphadenopathy or distant metastases (Figure 4).

## Discussion

Plexiform fibrohistiocytic tumor (PFHT) is a rare mesenchymal tumor that exhibits fibroblastic/myofibroblastic differentiation, mainly affecting children and young adults, with 87% of patients under the age of 37 years [4]. This tumor belongs to the group of so-called fibrohistiocytic neoplasms, but there is also evidence suggesting a myofibroblastic origin [11]. The tumor typically manifests as a slowly-growing, painless, solitary nodule of the dermal and subcutaneous tissues, with a predilection for the upper extremity [3]. The overlying skin is usually raised, firm with unclear borders; rarely, the lesion may present with a central depression, discoloration or ulceration [9,12]. These tumors are generally small in size and rarely exceed 3 cm in diameter [3]; however, giant lesions measuring up to 8 cm have also been reported [13].

PFHT is typically located in the deep dermis with extension into the adjacent subcutis; however, it can extend into the deeper subcutaneous tissue or underlying muscle [5]; therefore, adequate pre-operative radiological imaging is necessary [14]; Ghuman et al. used the MRI imaging, where most tumors had a plaque-like or infiltrative morphology extending in the subcutaneous tissues [15]. Differential diagnosis is based on pathologic examination and includes granulomatous inflammation, cellular neurothekoma, fibrous hamartoma of infancy, plexiform spindle cell naevus, plexiform neurofibroma and plexiform myofibroblastoma [5,9,15].

The congenital form of this tumor is extremely rare, with very few cases being reported in the literature so far [6–10]. Table 1 summarizes demographic data of the congenital PFHT cases reported in the English literature so far. In our case, the PFHT was present at birth and, thus, is classified as a congenital tumor; it was located over the foot instep, a relatively rare location, measured less than one cm in diameter, and was characterized by dermal and subcutaneous proliferation, which is typical for this type of tumors.

**Table 1.** Demographics of the reported congenital cases of plexiform fibrohistiocytic tumors.

Author	Sex	Age	Location	Size	Surgical treatment	Follow-up
Leclerc et al. 2005 [6]	Boy	2.5 months	Mandible	Unknown	Wide excision	Not reported
Jafarian et al. 2006 [10]	Girl	18 months	Chest	2.5 cm	Complete excision	11 months
Muezzinoglu et al. 2011 [7]	Girl	7 years	Waist	3 cm	Wide excision	26 months
Sandrini et al. 2011 [8]	Girl	3 years	Flank	0.5 cm	Excision	6 months
Nieto et al. 2018 [9]	Boy	Newborn	Foot	2.5 cm	Excision	Unknown
Demiri et al (current study)	Boy	4 years	Foot	0.8 cm	Wide excision	52 months

According to the literature, both acquired and congenital PFHTs are histologically composed of two main components: histiocyte-like and spindled fibroblast-like cells arranged in a plexiform pattern. Hemosiderin deposition is also a characteristic feature [2,5]. Nuclear atypia and cellular pleomorphism are usually absent and mitotic activity is low. Rarely, metaplastic bone formation has been reported in acquired cases [16].

Occasionally, PFHT may demonstrate aggressive behavior with local recurrence and, rarely, regional lymph node involvement and distant lung metastases [4,17–19]; no specific histological features have been clearly correlated with aggressive biologic behavior [4]. According to the literature, the reported local recurrence rate of acquired PFHTs varies from 12.5 to 40%, while local recurrences have been reported to occur within the first two years after surgical excision [3,11,16,19]. No local recurrence or distant metastases of congenital tumors have been reported so far.

Regarding the surgical approach, complete excision is the primary treatment, while the role of radiation and chemotherapy appears to be limited [15,20]. Although wide surgical resection is mandatory to reduce the risk of recurrence, excisional margins are not clearly defined in the reported studies [10]. Ghuman et al., in their recently published series on thirteen patients, reported that negative margins (defined as >0.1 cm from the specimen margin) were adequate to avoid local recurrence after a mean follow-up of 57 months [15]. Even in the most recent guidelines of the National Cancer Institute, there is no specifically defined surgical approach or suggested safe surgical margins; removal of the tumor along with some surrounding healthy tissue is only recommended [21]. Rahimi et al., pointed out that Mohs surgery is very useful to assure clear margins [22]; however, Mohs surgery seems unlikely to properly assess margins in PFHT, given its plexiform growth pattern which gives rise to spatially separated tumor nodules in histologic cross-sections. Moreover, PFHT does not occur in delicate anatomic locations that would justify performing an expensive and time-consuming Mohs procedure.

In our case, following the first incomplete resection, we decided to perform a 2 cm-margin wide excision around the existing scar extending deep to the underlying fascia, in order to avoid recurrence; a full-thickness skin graft was used to cover the surgical defect and provided stable coverage of the operated site over the foot instep. Indeed, after a 52 month-follow-up, no evidence of local recurrence or distant metastases was recorded. Our patient’s follow-up is still ongoing with yearly clinical examination of both the operated zone and regional lymph nodes, and chest x-ray performed once a year.

In conclusion, PFHT should be included in the differential diagnosis of an enlarging skin and subcutaneous tumor in children. Despite the absence of local recurrence and metastases in the reported congenital PFHT cases, and although the prognosis is generally favorable, this tumor shares the same histological features with the acquired form and thus, its clinical behavior is presently considered unpredictable. Therefore, wide local excision is recommended and high clinical vigilance is required on follow-up.
